# The Influenza Crisis at Birmingham

**Published:** 1918-11-30

**Authors:** Wm. Darby

**Affiliations:** Chairman, Birmingham Board of Guardians. Birmingham Union Offices, Edmund Street, Birmingham


*To the Editor of* The Hospital.

### The Influenza Crisis at Birmingham.

*To the Editor of* The Hospital.

Sir,—Will you kindly grant space in your columns for an appeal for volunteers to assist at the infirmary, Selly Oak, in nursing sufferers from influenza? Unfortunately, while the patients daily increase in number, a large number of the staff have been stricken down, and a crisis has arisen inasmuch as all efforts to secure additional nurses from the usual sources have failed. There are at the present time approximately 350 patients in the infirmary, of whom more than one-third are suffering from influenza, and more than two-thirds of the nursing staff are suffering from the same complaint.

Attempts have been made to ease the situation by the transfer of cases to other institutions of the Board, but owing to the widespread area of infection and to the largest hospital of the Guardians having been temporarily handed over to the military authorities, not much can be done in this direction.

I wish, therefore, to appeal to any ladies who have some knowledge of nursing to volunteer for service; possibly some of the ladies who have done such valuable work as V.A.D.s may be able to assist in the emergency. Offers of assistance should be made to Miss Bodley, matron of the infirmary.

The Guardians will appreciate and value any help you may be able to give them, either through the columns of your papers or in any other manner that may suggest itself to you.—Yours faithfully, Wm. Darby,

Chairman, Birmingham Board of Guardians.

Birmingham Union Offices, Edmund Street,
Birmingham, November 26, 1918.

[We hope many willing and capable hands will write to Miss Bodley and volunteer.—Ed., The Hospital.]

